# Analysis of the Interaction of Dp44mT with Human Serum Albumin and Calf Thymus DNA Using Molecular Docking and Spectroscopic Techniques

**DOI:** 10.3390/ijms17071042

**Published:** 2016-06-30

**Authors:** Zhongjie Xu, Youxun Liu, Sufeng Zhou, Yun Fu, Changzheng Li

**Affiliations:** 1 College of Life Science and Technology, Xinxiang Medical University, Xinxiang 453003, China; xcj029@163.com; 2 Department of Molecular Biology & Biochemistry, Xinxiang Medical University, Xinxiang 453003, China; liuyouxun@126.com (Y.L.); sufengzhou@xxmu.edu.cn (S.Z.); fuyun9801@163.com (Y.F.); 3 Henan Collaborative Innovation Center of Molecular Diagnostics and Laboratory Medicine, Xinxiang 453003, China

**Keywords:** Dp44mT, fluorescence quenching, molecular docking

## Abstract

Di-2-pyridylketone-4,4,-dimethyl-3-thiosemicarbazone (Dp44mT) exhibits significant antitumor activity. However, the mechanism of its pharmacological interaction with human serum albumin (HSA) and DNA remains poorly understood. Here, we aimed to elucidate the interactions of Dp44mT with HSA and DNA using MTT assays, spectroscopic methods, and molecular docking analysis. Our results indicated that addition of HSA at a ratio of 1:1 did not alter the cytotoxicity of Dp44mT, but did affect the cytotoxicity of the Dp44mT-Cu complex. Data from fluorescence quenching and UV-VIS absorbance measurements demonstrated that Dp44mT could bind to HSA with a moderate affinity (K_a_ = approximately 10^4^ M^−1^). CD spectra revealed that Dp44mT could slightly disrupt the secondary structure of HSA. Dp44mT could also interact with Ct-DNA, but had a moderate binding constant (K_EB_ = approximately 10^4^ M^−1^). Docking studies indicated that the IB site of HSA, but not the IIA and IIIA sites, could be favorable for Dp44mT and that binding of Dp44mT to HSA involved hydrogen bonds and hydrophobic force, consistent with thermodynamic results from spectral investigations. Thus, the moderate binding affinity of Dp44mT with HSA and DNA partially contributed to its antitumor activity and may be preferable in drug design approaches.

## 1. Introduction

Novel Fe and Cu chelators have emerged as a promising anticancer strategy [1]. The iron chelator di-2-pyridylketone-4,4,-dimethyl-3-thiosemicarbazone (Dp44mT) exhibits excellent antitumor activities in human pancreatic, melanoma, and neuroepithelioma xenografts, and reduces metastasis in a breast cancer model [2,3,4,5,6]. Mechanistic studies have revealed that Dp44mT has a variety of molecular targets and functions to inhibit the activities of ribonucleotide reductase and topoisomerase IIa, mobilize intracellular Fe, and prevent cellular Fe uptake from Tf [7,8]. Furthermore, Dp44mT can increase the expression of the potent metastasis suppressor N-myc downstream-regulated gene 1 and can be confined within the acidic lysosomal compartment to enhance autophagy [9,10]. However, no studies have examined the effects of the interaction between Dp44mT and biological molecules, such as proteins and nucleic acids. To gain insight into the mechanism of action, investigating the interaction of Dp44mT with proteins or DNA is necessary.

Human serum albumin (HSA) is an important component in serum and a carrier for exogenous and endogenous ligands. HSA/drug interaction has been realized to be a critical factor in absorption, metabolism, and excretion of the drug [11,12]. A study recently revealed that HSA both affected uptake and cytotoxicity of Dp44mT [13], indicating that the interaction of HSA with Dp44mT is an important factor mediating the drug’s activity. However, it is still unclear how the strength of the interaction influences uptake or cytotoxicity, and such factors should be considered during the drug design process. HSA contains three homologous domains (I, II, and III); each domain is composed of two subdomains (IA, IB, etc.) [14]. HSA has one tryptophan residue (Trp214), located within the hydrophobic binding pocket of subdomain IIA. The interactions of HSA with endogenous and exogenous ligands occur mostly in these domains.

DNA is the pharmacological target of many of drugs. Targeting DNA can regulate cell functions by modulating transcription or interfering with replication. Accordingly, the drug binding to DNA will artificially alter the functioning of DNA. These behaviors of the drug contribute to its biological activities in cell growth inhibition or control of a disease [15]. Dp44mT, as a promising candidate in cancer therapy, exhibits excellent biological activities, which may involve in the interaction with DNA, however, the study, especially in the mechanism of interactions between the drug and DNA, has not been conducted.

Spectral techniques are widely used to determine the interactions between biomacromolecules and ligands [16,17]. The objective of the present work was to obtain more detailed information regarding the specific binding domain of Dp44mT with HSA or DNA by employing computer-aided molecular docking and spectral techniques. The findings of our study may provide additional information with which to understand the antitumor activity of Dp44mT.

## 2. Results and Discussion

### 2.1. Human Serum Albumin (HSA) Affected the Cytotoxicity of Dp44mT and Its Copper Complex (Dp44mT-Cu)

**Figure 1:**
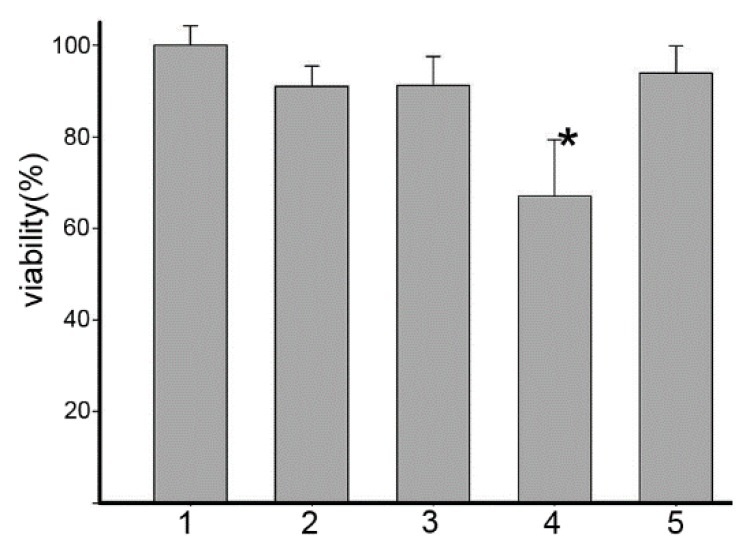
Inhibition of HepG2 cell proliferation by Dp44mT and its copper complex in the absence or presence of HSA. 1, control; 2, Dp44mT; 3, Dp44mT + HSA (1:1); 4, Dp44mT-Cu; and 5, Dp44mT-Cu + HSA (1:1). * *p* < 0.01 (*t*-tests).

HSA can enhance the uptake and cytotoxicity of Dp44mT; however, assays are typically performed in the presence of fetal calf serum (FCS) [13]. Therefore, we aimed to eliminate the possible effects of HSA on Dp44mT-mediated inhibition of proliferation by evaluating the effects of HSA on HepG2 cell proliferation in FCS-free medium. As shown in Figure 1, growth inhibition induced by Dp44mT in the presence of equimolar HSA was similar to that without HSA, in contrast to previous findings [13]. To further confirm the reliability of our procedure, we evaluated the Dp44mT-Cu complex in the same manner. Our findings showed that cell proliferation in the presence of the Dp44mT-Cu complex was significantly attenuated by the addition of HSA, indicating that the effects of HSA on uptake or cytotoxicity were drug-dependent.

### 2.2. UV-VIS Spectral Study of the Interaction between Dp44mT and HSA

**Figure 2:**
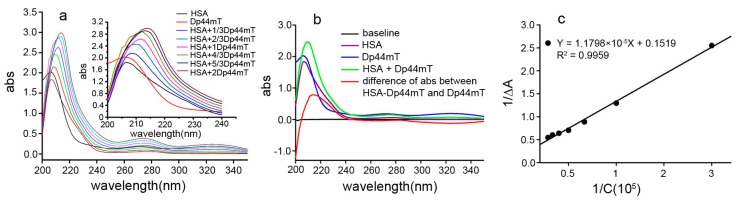
The interaction of Dp44mT with HSA. (**a**) Spectral changes upon addition of Dp44mT. The redshift around 214 nm is shown in the insert; (**b**) the difference spectrum between Dp44mT and HSA-Dp44mT; and (**c**) linear plots for 1/∆*A*_abs_ versus 1/[Dp44mT] at 288 K.

HSA is known to interact with Dp44mT; however, the binding constant has not yet been reported. Therefore, we determined the UV-VIS spectra of HSA in the absence or presence of Dp44mT to determine the binding affinity of Dp44mT with HSA. A peak shift was observed (Figure 2a). Moreover, in the difference spectrum (Figure 2b), an approximately 7 nm shift was observed when excess Dp44mT was added, further supporting the interaction between these components. The Benesi-Hildebrand equation are widely used for determining the binding constant [18,19]:

(1) $$\frac{1}{∆A_{abs}}=\frac{1}{\left[S\right]\left[L\right]\varepsilon K}+\frac{1}{\left[S\right]\varepsilon }$$

(2) $$K=\frac{y-\text{int }ercept}{slope}$$

where Δ*A*_abs_ is the change in the absorbance at 214 nm, *K* is the binding constant, [*S*] is the concentration of HSA, [*L*] is the concentration of Dp44mT, and ε (or Δε = ε_HSA-Dp44mT_ − ε_HSA_ − ε_Dp44mT_) is the extinction coefficient at 214 nm. Thus, a double reciprocal plot was generated to show absorbance changes at 214 nm as a function of the reciprocal concentration (Figure 2c). Clearly, there was a good linear relation between the changes in absorbance and concentration; thus, the K value can be calculated by the intercept divided by the slope and found to be approximately 1.28 × 10^4^ M^−1^. In general, values of the binding constant in the range of (1–15) × 10^4^ M^−1^ are considered moderate [20], consistent with previous results [13].

### 2.3. Fluorescence Quenching and Quenching Mechanism Analysis

**Figure 3:**
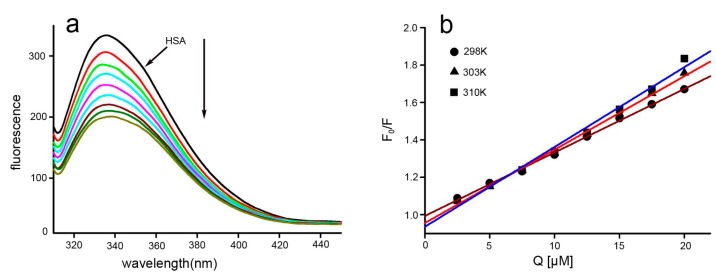
Fluorescence changes in HSA upon addition of Dp44mT and Stern-Volmer plot (pH 7.4, 298 K). (**a**) The fluorescence intensity deceased with increasing amounts of Dp44mT; and (**b**) Stern-Volmer plots for quenching of HSA fluorescence by Dp44mT at different temperatures. Down arrow: trend of fluorescence decreased.

Fluorescence spectroscopy is the most comprehensive method for studying protein-ligand interactions [21]. Thus, fluorescence spectroscopy was used to investigate the interactions between HSA and Dp44mT. Generally, the intrinsic fluorescence of HSA originates from its Trp214 residue alone. The fluorescence spectra of HSA following addition of difference concentrations of Dp44mT are shown in Figure 3a. The data revealed that the fluorescence intensity decreased markedly as the Dp44mT concentration increased and that the emission spectra were almost constant, regardless of HSA-Dp44mT complexation (1 nm redshift), indicating that Dp44mT could interact with HSA, but that the chromophore of HSA (Trp214) was not exposed to an obviously polar environment with the addition of Dp44mT. To gain insights into the nature of interactions between Trp214 of HSA and Dp44mT, the fluorescence quenching profile of HSA was modeled with the following Stern-Volmer equation [22]:

(3) $$\frac{F_{0}}{F}=1+K\text{sv}\left[Q\right]=1+Kq\tau_{0}\left[Q\right]$$

where *F*_0_ and *F* are the steady-state fluorescence intensities in the absence and presence of a quencher, respectively; [*Q*] is the concentration of the quencher; *K*_sv_ is the Stern-Volmer dynamic quenching rate constant; *τ*_0_ is the fluorescence lifetime of the protein without the quencher, the average life of a fluorescence molecule is 10^−8^ s [23]; and *K*_q_ is the quenching rate constant of the biomolecule. Plots were generated based on the Stern-Volmer Equation (3) (Figure 3b). The plots exhibited good linear relationships at the molar ratio of Dp44mT-HSA ≤2:1, suggesting the occurrence of a single type of quenching phenomenon, i.e., either static or dynamic quenching. In dynamic quenching, increasing the temperature results in faster diffusion and, hence, increased collision, thereby raising the quenching constant. In contrast, in static quenching, increasing the temperature weakens the stability of the formed complex and, hence, reduces the quenching constant [24]. Thus, additional experiments were conducted. The Stern-Volmer plots at different temperatures are shown in Figure 3b, and the *K*_sv_ and *K*_q_ values derived from Equation (3) at the three temperatures are presented in Table 1. Since these analyses were performed at λ_ex_ = 295 nm, the slopes increased as the temperature increased, indicating that interference with the Trp214 residue was involved in Dp44mT-mediated dynamic quenching of HSA fluorescence. Furthermore, *K*_q_ was much larger than the maximum scatter collision quenching constant, 2.0 × 10^10^ mol^−1^·s^−1^ (Table 1). Thus, the quenching mechanism could be explained by complex formation between Dp44mT and BSA, rather than dynamic collision.

**Figure 4:**
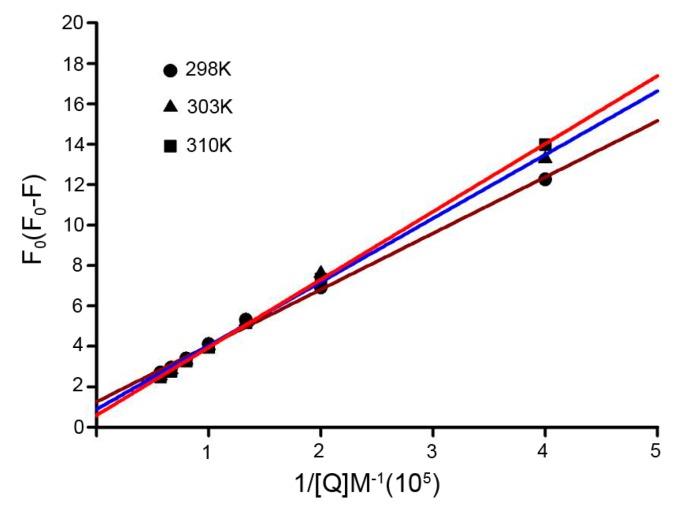
The modified Stern-Volmer plots used to obtain association constant of Dp44mT at different temperatures as previously described [16]. Conditions: [HSA] = 1.5 × 10^−5^, pH = 7.4.

Additionally, the quenching process was further analyzed using the following modified Stern-Volmer equation [24]:

(4) $$\frac{F_{0}}{F_{0}-F}=\frac{1}{f_{a}K_{a}}\frac{1}{\left[Q\right]}+\frac{1}{f_{a}}$$

where, for our study, *F*_0_ and *F* are the fluorescence intensity in the absence and presence of the quencher, respectively; *K_a_* is the effective quenching constant for the accessible fluorophores, which is analogous to the association binding constants for the quencher-acceptor system; [*Q*] is the concentration of the quencher; and *f*_a_ is the fraction of accessible fluorescence. As shown in Figure 4, the curves of *F*_0_/(*F*_0_ − *F*) versus [*Q*]^−1^ were linear when calculated according to quencher concentrations. The corresponding parameters are presented in Table 2. Moreover, the decreasing trend of *K*_a_ indicated that the binding of Dp44mT to HSA was reduced as the temperature increased.

### 2.4. Binding Constants and Binding Sites

The equilibrium process of HSA and Dp44mT can be described by the following equation, and the important binding parameters, including the association constant (*K*_b_) and number of binding sites (n), could be calculated through the plots [25]:

(5) $$\log\left(\frac{F_{0}}{F_{0}-F}\right)=logK_{b}+nlog\left[Q\right]$$

**Figure 5:**
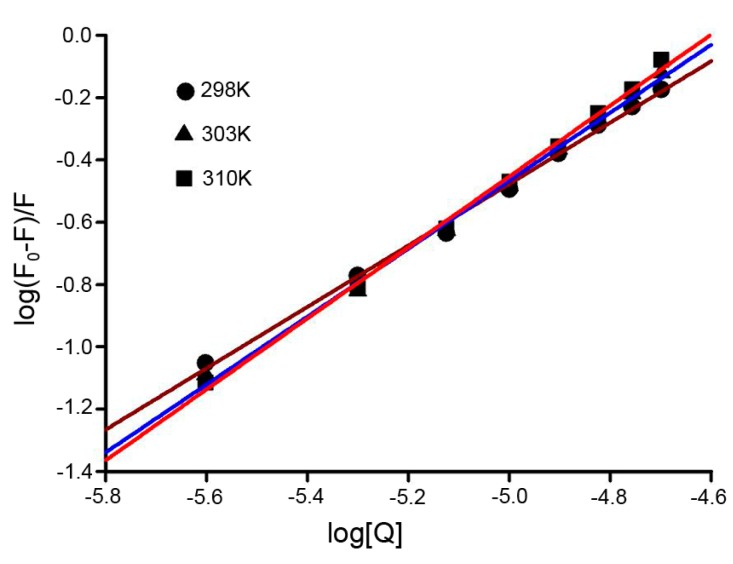
Plots of log[(*F*_0_ − *F*)/*F*] versus log[*Q*] used to assess the quenching effect of Dp44mT on HSA fluorescence as reported method [16]. The temperature is as indicated in the figure. *C*_HSA_ = 1.5 × 10^−5^ M; pH 7.4; λ_ex_ = 295 nm, λ_em_ = 300–450 nm.

*K*_b_ and n were obtained from the intercept and slope of the curve. Thus, the plots of log[(*F*_0_ − *F*)/*F*] versus log[*Q*], based on Equation (5) were generated to determine the binding parameters (Figure 5). The values of *K*_b_ and n were evaluated and are presented in Table 3. The number of binding sites (*n*) was approximately 1, indicating that there was one HSA binding site for Dp44mT. The value also showed that *K*_b_ increased as temperature increased, consistent with the aforementioned trend of *K*_sv_.

### 2.5. Thermodynamic Parameters and Nature of the Binding Forces

**Figure 6:**
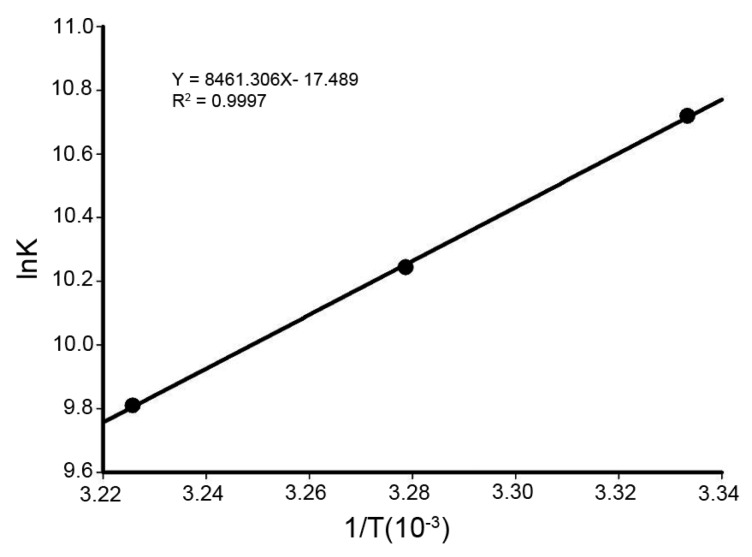
Van’t Hoff plot used to predict the interaction between HSA and Dp44mT as described method [16]. Conditions: *C*_HSA_ = 1.5 × 10^−5^ M, pH 7.4; λ_ex_ = 295 nm, λ_em_ = 300–450 nm.

The interaction between biomacromolecules and small molecules can be through hydrogen bonds, electrostatic interaction, hydrophobic force, and van der Waals forces. The manner of the action involved in binding can be also reflected from thermodynamic parameters: Δ*H* > 0 and Δ*S* > 0 indicated a hydrophobic interaction, Δ*H* < 0 and Δ*S* < 0 hinted hydrogen bonding and van der Waals interactions, and Δ*H* < 0 and Δ*S* > 0 implied electrostatic interactions [26,27]. Generally the thermodynamic parameters were deduced from the van’t Hoff equation (6):

(6) $$\ln K=\frac{-∆H}{RT}+\frac{∆S}{R}$$

where *K* is the binding constant, *T* is the absolute temperature, and *R* is the universal gas constant. Δ*H* and Δ*S* were obtained from the slope and intercept of the linear van’t Hoff plot (shown in Figure 6). The free energy change (Δ*G*) was estimated using the following formula:

Δ*G* = Δ*H* − *T*Δ*S* (7)

The values of Δ*H*, Δ*S*, and Δ*G* for Dp44mT binding to HSA are listed in Table 4. Based on the view of Ross and Subramanian, negative Δ*H* and Δ*S* values implied that the interaction between Dp44mT and HSA occurred via hydrogen bonding and van der Waals interactions, consistent with a study of a Dp44mT analog [16]. The negative value of Δ*G* also revealed that the interaction process was spontaneous.

### 2.6. Energy Transfer between Dp44mT and HSA

**Figure 7:**
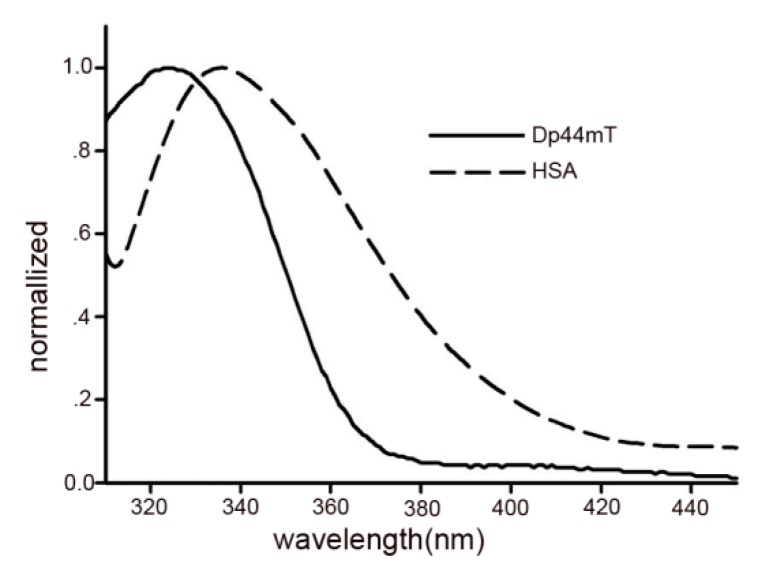
Overlap of the fluorescence spectrum of HSA and absorption spectrum of Dp44mT indicated that FRET occurred [16].

Fluorescence resonance energy transfer (FRET) is widely used to determine the distance between donor and acceptor [28] and occurs when emission spectrum of a donor (fluorophore) overlaps with the absorption spectrum of a bound ligand (acceptor). The feature of fluorescence of Trp214 (donor) in HSA and bound Dp44mT (acceptor), as well as overlap of the absorption spectrum with fluorescence spectrum (Figure 7), made possible the determination of the distance between the donor and acceptor using FRET. The efficiency of energy transfer can be used to evaluate the distance between Dp44mT and the Trp214 residue. The relationship between the distance and efficiency of FRET is described in the following equation:

(8) $$E=1-\frac{F}{F_{0}}=\frac{R_{0}^{6}}{R_{0}^{6}+r^{6}}$$

where *F* and *F*_0_ are the fluorescence intensity of HSA in the presence or absence of the acceptor, respectively; r is the distance between the acceptor and donor; and *R*_0_ is the critical distance for 50% energy transfer, which can be calculated using the following:

(9) $$R_{0}=8.8\times 10^{-25}K^{2}N^{-4}\Phi J$$

where *K*^2^ is the spatial orientation of the transition dipoles of the donor and acceptor (*K*^2^ = 2/3 for a random orientation), *N* is the average refractive index of medium (*N* = 1.336), Φ is the fluorescence quantum yield of the donor (*Φ* = 0.15) [29], and *J* is a factor describing the overlap between the emission spectrum of the donor and the absorption spectrum of the acceptor. *J* is given by the following equation:

(10) $$J=\frac{\Sigma F\left(\lambda \right)\varepsilon \left(\lambda \right)\lambda^{4}∆\lambda }{\Sigma F\left(\lambda \right)∆\lambda }$$

where *λ* is the wavelength. To calculate the distance (*r*), *E* and *J* values are required; these can be obtained by calculating fluorescence data and overlapped areas between the HSA emission spectrum and the absorption spectrum of Dp44mT. The efficiency of FRET to be used in Equation (8) was estimated by measuring the fluorescence at equal protein/ligand concentrations, as previously described [30]. Based on Equations (8)–(10), *J*, *R*_0_ (nm), *E*, and *r* (nm) were calculated (Table 5). The calculated *r* value was 2.03 nm (*R*_0_ = 2.40 nm). Clearly, the distance between HSA and Dp44mT was less than 8 nm and within 0.5 *R*_0_ < *r* < 1.5 *R*_0_, indicating that the energy transfer from Trp214 to Dp44mT occurred with high possibility [31]. Thus, the calculated results were well predicted by Föster’s nonradioactive energy transfer theory [32] and further verified that Dp44mT was likely to be located in domain IIIA or I of HSA [33].

### 2.7. Conformational Investigation by Synchronous Fluorescence

**Figure 8:**
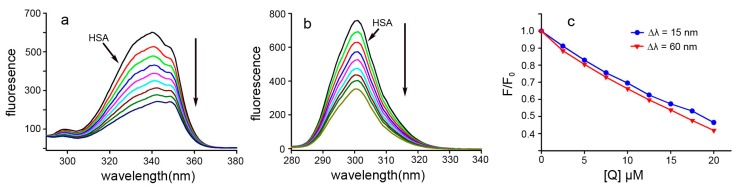
Effects of Dp44mT on the synchronous fluorescence spectra of HSA at 298 K. (**a**) Δλ = 60 nm; (**b**) Δλ = 15 nm; and (**c**) the effects of Dp44mT on Trp and Try fluorescence. *C*_HSA_ = 15.0 μM. The trend of fluorescence quenching was similar previous reported [16]. Down arrow: trend of fluorescence decreased.

Synchronous fluorescence spectra can provide information regarding the molecular environment in the vicinity of the chromophore molecules [34]. In this method, by using different D-values (Δλ) between the excitation and emission wavelengths, fluorescence contributors from different aromatic amino acids in the protein can be identified. The characteristic information for the Tyr and Trp residues can be provided when the scanning interval Δλ is fixed at 15 and 60 nm, respectively [35]. Synchronous fluorescence spectra of Dp44mT at Δλ = 60 and 15 nm are shown in Figure 8. Importantly, there was no significant shift in the maximum emission wavelength at Δλ = 15 nm (Figure 8b) or 60 nm (Figure 8a), implying that the interaction of Dp44mT and HSA could not affect the microenvironment around the Tyr or Trp residues. Furthermore, there was a slight difference in the quenching efficiency of the fluorophore residues by Dp44mT; the fluorescence intensity quenching of Trp residue was decreased by approximately 58% (Figure 8c), whereas that for the Tyr fluorophore was decreased by a lesser amount.

### 2.8. Circular Dichroism (CD) Spectra

The changes in the secondary structure of HSA were also found with the addition of the drug, which can be determined by CD spectroscopy, thereby CD spectra were measured in the absence or presence of different concentrations of Dp44mT (Figure S2). In the HSA CD spectrum, a characteristic peaks of the α-helix structure (two negative bands at 208 and 222 nm) was observed as in a previous report [13]. In the presence of different concentrations of Dp44mT, a slight reduction in negative ellipticity was observed, indicating that the α-helix content of the protein was decreased (Figure S2). The contents of secondary structure can be calculated based on experimental data [36], but it was limited due to larger errors. Thus, we used K2D2 software (Helsinki University of Technology, Espoo, Finland) to simulate the experimental data from CD spectroscopy [37]. The results indicated that α-helix and β-sheet contents of HSA in the absence of Dp44mT were 43.94% and 10.56%, respectively (Figure S2). Following the addition of Dp44mT, the α-helix and β-sheet contents were changed to 39.07% and 10.04%, respectively, at the molar ratio of drug/protein (d/p) = 2, representing an approximately 5% decrease in α-helix content, in contrast to a previous report [13].

### 2.9. Interaction of Dp44mT with Ct-DNA

**Figure 9:**
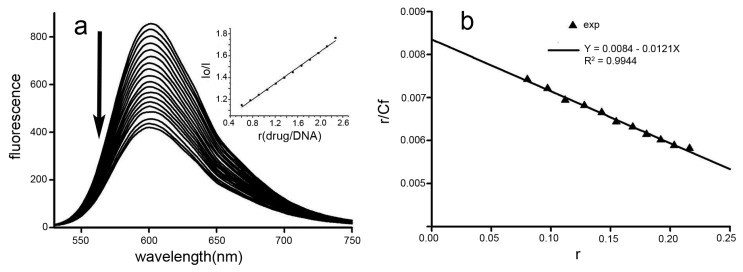
Fluorescence quenching spectra of EB-Ct-DNA by Dp44mT. (**a**) Fluorescence decreased with increasing Dp44mT, the insert showing that the I_0_/I increased with concentration of Dp44mT; and (**b**) Scatchard plot used to determine the association constant K_EB_ as described [38]. Down arrow: trend of fluorescence decreased.

Small ligand molecules bind to DNA and artificially alter and/or inhibit the functioning of DNA. Interaction studies between Dp44mT and DNA have improved our understanding of the interaction mechanisms. To investigate the mode of binding of Dp44mT to DNA, competitive binding experiments were carried out. The fluorescent emission spectra of ethidium bromide (EB; 2.5 μM) bound to DNA (16.5 μM) in the absence and presence of Dp44mT are shown in Figure 9. EB is a conjugate planar molecule and can be intercalated into the base pairs of double-stranded DNA, contributing to its enhanced fluorescence. Dp44mT does not show appreciable fluorescence in the spectral region studied, both in the free and DNA-bound states. The fluorescence intensities at 602 nm were decreased with increased Dp44mT concentrations, as shown in Figure 9a (insert: changes in fluorescence quenching with the Dp44mT/DNA ratio). These results suggested that Dp44mT had weak intercalating ability. To obtain insights into the nature of Dp44mT-DNA binding, the binding of EB to DNA was investigated in the absence and presence of Dp44mT using the Scatchard equation [38]:

(11) $$\frac{r}{C_{f}}=K\left(n-r\right)$$

where *r* is the ratio of bound EB to total DNA, *C*_f_ is the concentration of free EB, *n* is the number of binding sites per nucleic acid, and *K* is the intrinsic binding constant for EB. According to Equation (11), the calculated *K* and *n* values were 1.21 × 10^4^ M^−1^ and 0.694, respectively, indicating weaker binding to Ct-DNA (Figure 9b).

### 2.10. Molecular Docking

**Figure 10:**
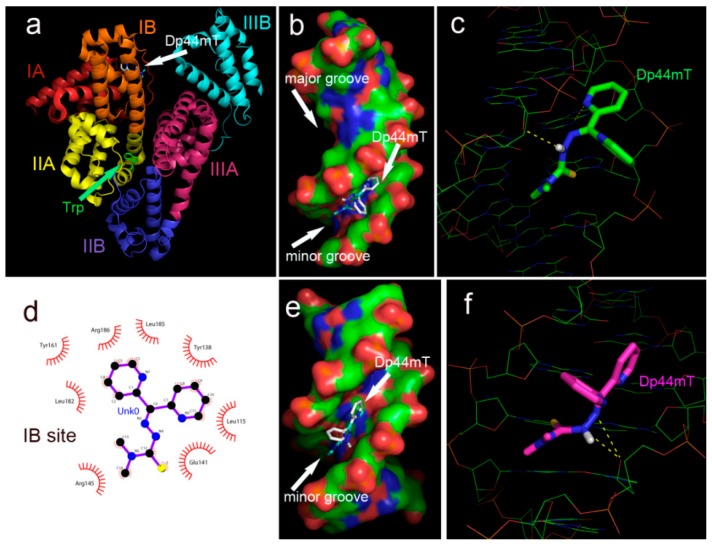
The interaction of Dp44mT with HSA and DNA. (**a**) Dp44mT located at the IB site; (**b**) Dp44mT located in the minor groove of TA-enriched DNA; (**c**) interaction of Dp44mT with the adjacent nucleoside of TA-enriched DNA; (**d**) interaction of Dp44mT with environmental residues (in the figure Unk0 = Dp44mT); (**e**) Dp44mT located in the minor groove of GC-enriched DNA; and (**f**) interaction of Dp44mT with the adjacent nucleoside of GC-enriched DNA. The eye-like curved dark red lines represent the hydrophobic residues in contact with Dp44mT as previously described [16].

The interactions between Dp44mT and HSA, as determined from spectral data, promoted us to examine the structural basis of such interactions. To this end, we performed molecular docking, which is widely used to predict the interactions of small molecules with biomolecules. The crystal structures of HSA (PDB ID: 2bxd, 2bxg, and 4g03) were obtained from the RCSB Protein Data Bank. Dp44mT was individually docked into Sudlow’s sites I and II of HSA, and the simulating affinity energies for Dp44mT at IIA and IIIA with HSA were −7.4 and −7.1 kcal/mol, respectively; preferential binding was not supported. The interactions of Dp44mT with Trp214 and other amino acids at binding sites are shown in Figure S3. Since the chosen structures of HSA were bound with drug ligands (2bxd and 2bxg), to avoid possible docking errors, the “naked” HSA structure (4g03) was used for further docking analyses to identify favorable binding sites other than sites IIA and IIIA. Interestingly, Dp44mT could also bind at the IB site with a −7.5 kcal/mol affinity energy based on the results from random simulation, indicating that the IB site may be a potent binding pocket for Dp44mT. The IB site is considered the third major drug-binding region [39], and Dp44mT may have a similar mechanism of action, even in the absence of crystal structural evidence. The favorable location and interaction of Dp44mT with HSA at the IB site are presented in Figure 10a,d, respectively. The results, both from spectral and molecular docking studies, indicated that the interaction of Dp44mT with HSA exhibited moderate affinity, and we speculated that Dp44mT could favor both the translocation and dissociation of HSA. Thus, the moderate binding affinity of Dp44mT to biological molecules may be an important factor mediating the antitumor activity of this molecule.

Following a similar procedure, Dp44mT was docked onto DNA (PDB ID: 1al9; Figure S3c). The simulated affinity energy for Dp44mT with double-stranded DNA was −7.1 kcal/mol, consistent with the results of fluorescence titration of EB-DNA. To determine whether Dp44mT tended to bind to a specific DNA sequence, DNA crystal structures enriched in GC (PDB ID: 4r49) or TA (PDB ID: 5et9) were used for further docking studies, and the simulated results are shown in Figure 10b,c,e,f. In both cases, Dp44mT bound to the minor groove of DNA, and the affinity energies for GC- and TA-enriched DNA were −6.2 and 6.4 kcal/mol, respectively. Thus, there was no evidence for preferential binding of Dp44mT to a specific sequence.

## 3. Experimental Section

### 3.1. Materials

All reagents used in this study were AR grade. The Ct-DNA was purchased from Sigma. The concentration of Ct-DNA of stock solution was calculated based on absorption (ε_260_ = 6600 L·mol^−1^·cm^−1^ at 260 nm). The Dp44mT was prepared by refluxing di-2-pyridylketone with 4,4,-dimethyl-3-thiosemicarbazide (Sigma, Shanghai, China) in ethanol. The mass spectrum is shown in Figure S1.

### 3.2. Proliferation Assay

Ten millimoles of stock solution of Dp44mT (in 80% DMSO) was diluted to the required concentration with culture medium prior to use. HSA was dissolved in phosphate-buffered saline (PBS) and sterilized by filtration. HepG2 cells in the exponential-phase were collected and seeded equivalently in 96-well plates, after 6 h of incubation in RPMI-1640 medium supplemented with 10% fetal calf serum (FCS) and antibiotics. The medium was then removed, and the cells were washed with PBS. One hundred microliters of medium without FCS was then added to the cells, along with varying amounts of Dp44mT. The cells were then incubated for 8 h. the cellular viability was determined as previous reported [16].

### 3.3. Spectrophotometric Study

The absorption spectrum of Dp44mT (15 μM, in Tris-HCl buffer, pH 7.4) was measured on a Shimadzu UV-2450 spectrophotometer (Shimadzu Co., Ltd., Suzhou, China).

### 3.4. Fluorescence Study

Fluorescence titration of HSA by Dp44mT was conducted on an RF-5301 spectrofluorophotometer (Shimadzu Scientific Instruments, Kyoto, Japan). The protocol was the same as previously described, except the excitation wavelength was at 295 nm [16]. Briefly, 4.0 mL HSA solution (15 × 10^−5^ M) was manually titrated by successive addition of 1 μL of 10 mM Dp44mT. Each addition was mixed well, and followed by 5 min incubation, then the emission spectra were measured on the spectrofluorophotometer at 298 K. Fluorescence spectra at 303 or 310 K were also recorded in the same way. Synchronous fluorescence spectra were measured by simultaneously scanning at a fixed wavelength interval (Δλ = λ_em_ − λ_ex_ = 15 or 60 nm) and the procedure was the same as the above-motioned titration.

The fluorescence quenching of EB-Ct-DNA by Dp44mT was measured using the same instrument. Ten millimoles of phosphate buffer with 1 mM EDTA (pH 7.4) was used in the study. The Ct-DNA was dialyzed in phosphate buffer for three days with continuous agitation at 5 °C until the OD_260/280_ was greater than 1.9. Next, the mixture of 16.5 μM Ct-DNA with 2.5 μM EB (4 mL) was titrated by successive addition of 1 μL of 10 mM Dp44mT at intervals of 5 min. The emission spectra were recorded on the RF-5301 spectrofluorophotometer (excitated at 520 nm).

### 3.5. Measurement of CD Spectra

The changes of HSA in secondary structure were measured on a Chirascan (Applied Photophysics Ltd., Surrey, UK) in a 1.0 cm quartz cell. The CD spectra (200–250 nm) in the presence or absence of Dp44mT were recorded with a scan rate of 50 nm/min. 100 μL HSA (1.0 × 10^−5^ M in PBS) were titrated by varied volume of Dp44mT (0, 1, or 2 μL of 1 mM solution, which prepared by diluting 10 mM stock solution in DMSO with water). In the measurements the DMSO concentration was less than 0.5%. The average data were recorded from three scans and the absorption of the buffer solution (PBS) was manually subtracted from each spectrum.

### 3.6. Molecular Docking

Docking studies were performed by using AutoDock 4.0 software (The Scripps Research Institute, La Jolla, CA, USA) [40]. The structural data of HSA (PDB entry code: 2bxd, 2bxg, and 4g03) or DNA (DNA PDB entry codes: 1a19, 4r49, and 5et9) were obtained from the RSCB protein Data Bank. The structure of Dp44mT was generated from ChemDraw (PerkinElmer, Waltham, MA, USA). The orientation and conformation of Dp44mT in binding pocket were determined by the AutoDock program (The Scripps Research Institute, La Jolla, CA, USA). Accordingly, the PDBQT files were created by employing AutoDockTools. The grid box (22 × 24 × 28) was set according to the binding site for ibuprofen or warfarin. For random simulation, the grid box was enlarged. The crystallographic conformation of ibuprofen or warfarin in HSA was utilized as a reference to evaluate the accuracy of the predicted model [41]. PyMol and LigPlot were used to display the conformation and interaction [42,43].

## 4. Conclusions

In this study, we used spectroscopic and molecular modeling methods to investigate the interaction between Dp44mT and HSA. The results showed that Dp44mT was associated with HSA at three well-defined binding sites as other drugs via hydrogen bonds, hydrophobic forces, and van der Waals contacts as a spontaneous process. The docking results demonstrated that the IB domain seemed to be the preferential site for Dp44mT, with a slightly higher affinity energy. Dp44mT could also bind to the warfarin and ibuprofen binding sites (sites IIA and IIIA) with similar binding affinities. However, it should be noted that random simulation may be more reliable because there was no prerequisite for limited sites in the docking procedure. We speculated that the IB site could be a favorable site for Dp44mT, even though other sites could not be excluded. The nonradioactive energy transfer from HSA to Dp44mT at the IB (or IIIA) site occurred with high probability. The distance (r) calculated based on FRET between Dp44mT and the HSA Trp214 residue was closer to that measured by PyMol (approximately 2.35 nm). There was no peak shift in the fluorescent spectra, suggesting that the microenvironment of Tyr and Trp residues did not change. The slight changes in CD spectra suggested that the secondary structure of HSA may have been disrupted after binding with Dp44mT. These results supported that the Dp44mT binding site could be site IB or IIIA. The binding constant of Dp44mT to HSA or DNA was approximately 10^4^, in accordance with results of molecular docking analysis. In summary, this study provided valuable insights into the binding mechanism of Dp44mT and HSA, and the thermodynamic parameters obtained from our study could provide a better understanding of the effects of Dp44mT during the course of its transportation and distribution in the blood. Importantly, this moderate interaction between Dp44mT and HSA could contribute to the excellent antitumor activity of Dp44mT and could be applied during drug design processes.

## Figures and Tables

**Table 1:** The Stern-Volmer quenching constant *K_sv_* and bimolecular quenching rate constant *K_q_* of the HSA-Dp44mT system at different temperatures were deduced from Figure 3b as described [16]. λ_ex_ = 295 nm, λ_em_ = 290–500 nm.

pH	*T* (K)	*K*_sv_ (10^4^ M^−1^)	*K*_q_ (10^12^ M^−1^)	*R*^2^
7.4	290	3.39	3.39	0.9988
	300	3.92	3.92	0.9962
	310	4.26	4.26	0.9922

**Table 2:** Modified Stern-Volmer association constant (*K*_a_) deduced from Figure 4 [16]. λ_ex_ = 295 nm, λ_em_ = 300–450 nm.

pH	*T* (K)	*K*_sv_ (10^4^ M^−1^)	*R*^2^
7.4	298	4.52	0.997
	303	2.81	0.997
	310	1.82	0.999

**Table 3:** Binding constant *K*_a_ and the number of binding sites *n* at different temperatures were deduced from Figure 5 as described [16]. λ_ex_ = 295 nm, λ_em_ = 300–450 nm.

pH	*T* (K)	*K*_a_ (10^4^ M^−1^)	*n*	*R*^2^
7.4	298	2.82	1.112	0.997
	303	9.68	1.027	0.992
	310	17.46	1.019	0.995

**Table 4:** Thermodynamic parameters obtained for the Dp44mT-HSA interaction as described [16]. Conditions: pH 7.4, λ_ex_ = 280 nm, λ_em_ = 290–450 nm.

pH	*T* (K)	Δ*G* (kJ/mol)	Δ*H* (kJ/mol)	Δ*S* (J/mol·K)
7.4	298	−27.01	−70.34	−145.41
	300	−26.28		
	310	−25.26		

**Table 5:** The calculated values of *J*, *r*, *R*_0_, and *E* of HSA with Dp44mT.

System	*J* (cm^3^·L·mol^−1^)	*E* (%)	*R*_0_ (nm)	*r* (nm)
HSA-Dp44mT	8.38×10^13^	0.341	2.40	2.06

## Supplementary Materials

Supplementary materials can be found at http://www.mdpi.com/1422-0067/17/7/1042/s1.

## Abbreviations

HSA	Human Serum Albumin
Dp44mT	Di-2-pyridylketone-4,4,-dimethyl-3-thiosemicarbazone
FRET	Fluorescence Resonance Energy Transfer
CD	Circular Dichroism
Tf	Transferrin
