# Correction: Zhongjie Xu, et al. Analysis of the Interaction of Dp44mT with Human Serum Albumin and Calf Thymus DNA using Molecular Docking and Spectroscopic Techniques. *Int. J. Mol. Sci.* 2016, *17*, 1042

**DOI:** 10.3390/ijms17111915

**Published:** 2016-11-16

**Authors:** Zhongjie Xu, Youxun Liu, Sufeng Zhou, Yun Fu, Changzheng Li

**Affiliations:** 1 College of Life Science and Technology, Xinxiang Medical University, Xinxiang 453003, China; xcj029@163.com; 2 Department of Molecular Biology & Biochemistry, Xinxiang Medical University, Xinxiang 453003, China; liuyouxun@126.com (Y.L.); sufengzhou@xxmu.edu.cn (S.Z.); fuyun9801@163.com (Y.F.); 3 Henan Collaborative Innovation Center of Molecular Diagnostics and Laboratory Medicine, Xinxiang 453003, China

## 1. Correction

As a result of a recent letter to the editor [1], we would like to make several corrections to our manuscript [2].

We would like to correct the sentence on page 1: “no studies have examined the effects of the interaction between Dp44mT and biological molecules, such as proteins and nucleic acids”. This sentence should read: “information related to the effects of the interaction between Dp44mT and biological molecules such as human serum albumin (HSA) or DNA has not yet been fully and systematically studied”.

Furthermore, we provide information on the correctness and purity of the synthesized Dp44mT.

## 2. Results and Discussion

We used commercially available di-2-pyridylketone and *N*’,*N*’-dimethyl-3-thiosemicarbazide to synthesize Dp44mT via a condensation reaction. The reaction product (Dp44mT) was subjected to a specific re-crystallization in order to remove trace amount of *N*’,*N*’-dimethyl-3-thiosemicarbazide. The purity after recrystallization was assessed by thin layer chromatography (TLC) and HPLC. The results show that high purity of more than 98.5% was achieved (Figure C1 and Figure C2).

To characterize the structure of the synthesized Dp44mt, ^1^H-NMR, ^13^C-NMR and element analysis were performed. The ^1^H-NMR spectrum (Figure C3) showed that the product was of high purity and suitable for biological research (water peak from *d*_6_-DMSO was observed in the spectrum): ^1^H-NMR (*d*_6_-DMSO, ppm): 14.94 (s, NH), 8.86 (d, H, *J* = 4 Hz), 8.61 (d, H, *J* = 4 Hz), 7.99 (dt, 2H), 7.93 (d, H, *J* = 8 Hz), 7.60 (m, H, *J* = 8 Hz), 7.49 (dd, H, *J* = 8 Hz), 3.40 (s, 6H) (http://webspectra.chem.ucla.edu/NotesOnSolvents.html). The peaks of the protons on pyridines at the same positions in Dp44mT did not overlap, indicating that those protons were in slightly different environments. To support this speculation, the ^13^C-NMR spectrum was also recorded (Figure C4), and all carbon signals were found except for the peaks of carbons connected to nitrogen, which coincided with the DMSO signal: ^13^C-NMR (*d*_6_-DMSO, ppm): 180.48, 156.28, 151.62, 148.76, 148.20, 143.07, 138.30, 137.73, 127.14, 124.94, 124.39, 124.11 (the two carbons connected to nitrogen (N(CH_3_)_2_ overlapped with DMSO (Figure C4)). Both ^1^H-NMR and ^13^C-NMR spectra supported that the structure of Dp44mT synthesized was correct. Furthermore, elemental analysis confirmed that the synthesized Dp44mT was as expected and of high purity: C_14_H_15_N_5_S: Cal. (found) (%): C: 58.92 (58.80); H: 5.30 (5.25); N: 24.54 (24.62); S: 11.24 (11.30).

## 3. Materials and Methods

### 3.1. General Information

All the reagents and solvents used were of AR grade. The Dp44mT was prepared by reacting di-2-pyridylketone with 4’4’-dimethyl-3-thiosemicarbazide (Sigma, Shanghai, China) in absolute ethanol and allowed to cool [3]. The resulting precipitate was collected by suction filtration and washing with cold ethanol.

### 3.2. Purity Assessment and Structure Identification

To achieve high quality, the precipitates were re-crystallized in absolute ethanol under less saturating conditions. Upon achieving room temperature, moderate amounts of water were added, and the solution was then placed at −20 °C. The formed crystalline Dp44mT was filtered with ice-cold ethanol and dried in a vacuum desiccator. The purity assessments indicated that the Dp44mT was of high purity (98.5%).

Purity assessment via TLC showed only a single spot on the silica gel (Figure C1). Solvents: CHCl_3_/MeOH = 16:1 (the reactants and Dp44mT as indicated). HPLC was performed on a LC-20AT HPLC (Shimadzu Corpation, Kyoto, Japan) with the following protocol: Gradient: 15%–80% solvent B within 15 min, following increased to 100% in 10 min, and decreased to 15% in 5 min. Solvent A: water plus 0.1% TFA; solvent B: acetonitrile plus 0.1% TFA (Figure C2). NMR spectra were recorded on an Ascend^TM^ 400 spectroscope (Bruker, Fällanden, Switzerland) operating at 400 MHz. Elemental analysis was carried on a CHN2400 Perkin-Elmer (Waltham, MA, USA).

## Figures and Tables

**Figure C1:**
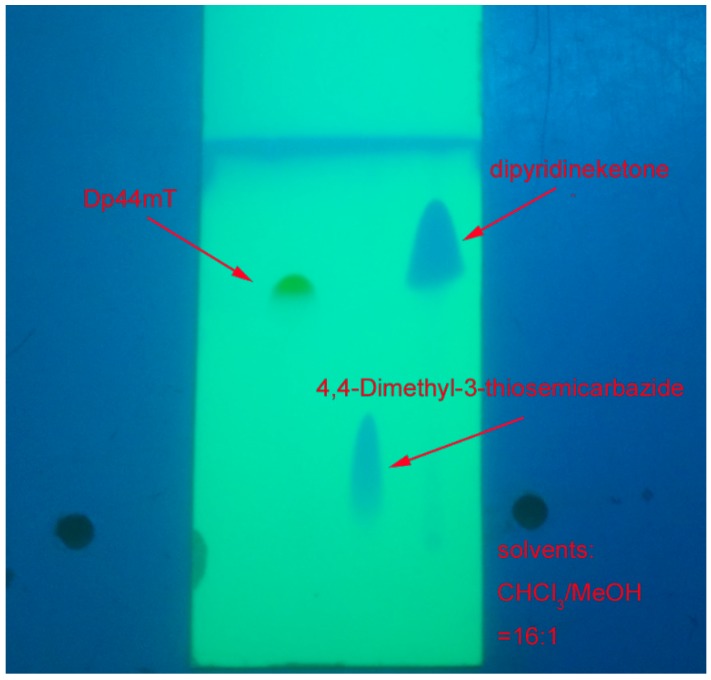
Thin layer chromatography (TLC) of synthesized Dp44mT.

**Figure C2:**
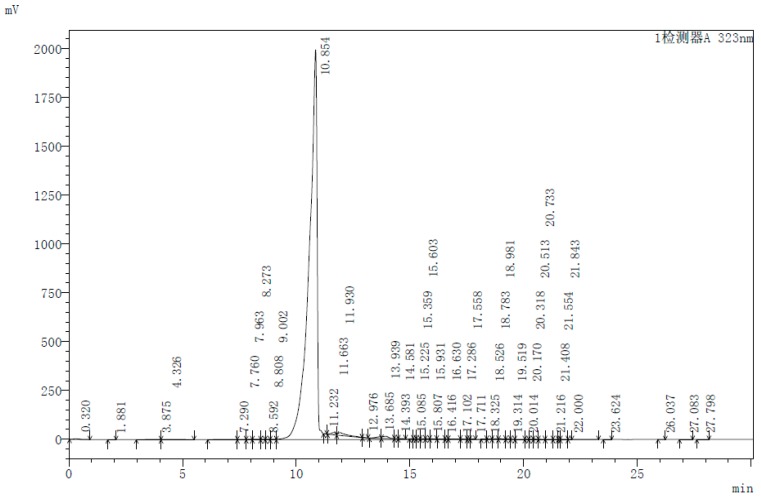
HPLC chromatogram of synthesized Dp44mT.

**Figure C3:**
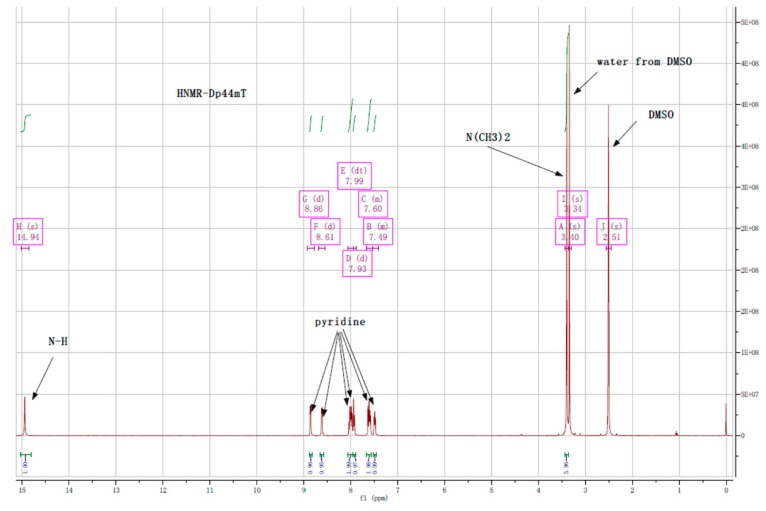
^1^H-NMR of Dp44mT and peak assignment.

**Figure C4:**
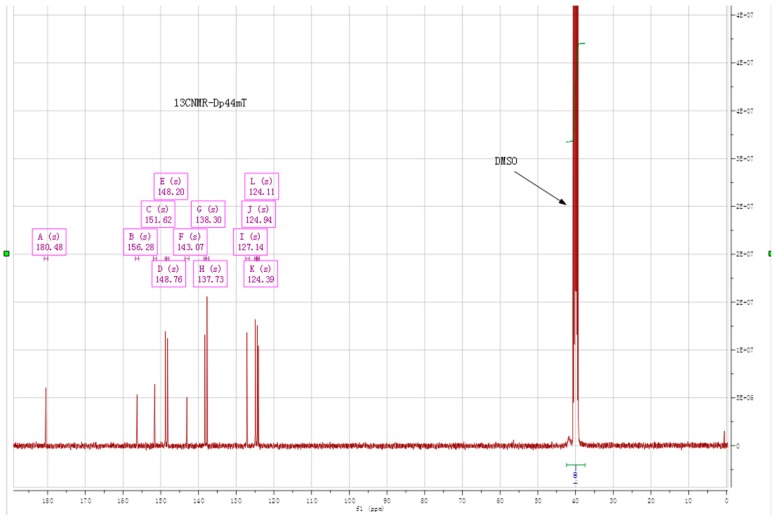
^13^C-NMR of Dp44mT and peak assignment.
