# Supplementary material for: Analysis of the Interaction of Dp44mT with Human Serum Albumin and Calf Thymus DNA Using Molecular Docking and Spectroscopic Techniques
Source: Int J Mol Sci. 2016 Jun 30;17(7):1042. doi: 10.3390/ijms17071042 (PMC4964418; doi:10.3390/ijms17071042)
Supplement: Supplementary file 1 [file ijms-17-01042-s001.pdf]

Supplementary Materials: Analysis of the Interaction of Dp44mT with Human Serum Albumin and Calf Thymus DNA Using Molecular Docking and Spectroscopic Techniques

Zhongjie Xu, Youxun Liu, Sufeng Zhou, Yun Fu and Changzheng Li

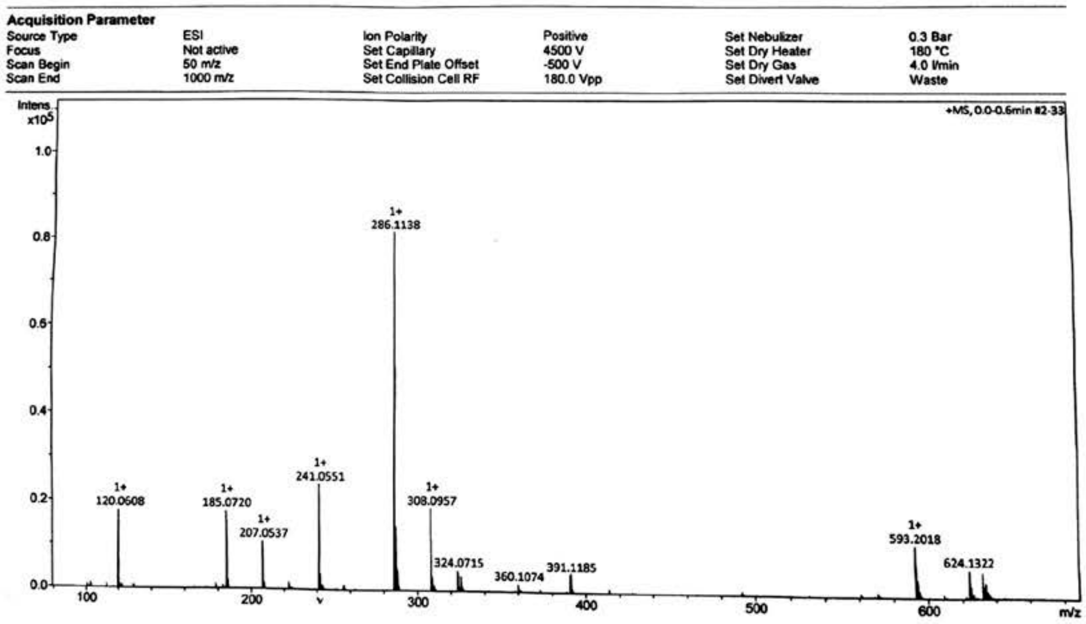

Figure S1. The mass spectrum of Dp44Mt.

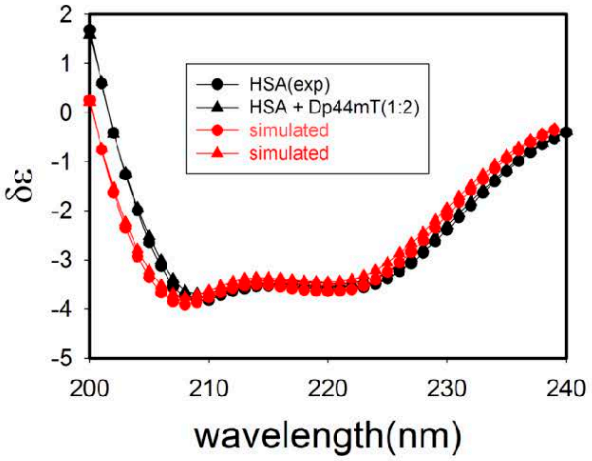

Figure S2. The CD spectra of interaction of Dp44Mt and HSA: Dp44mT and HSA: Dp44mT concentration were 0.0 and 20 μM, respecively. C_{HSA} = 10 μM, pH = 7.4 and T = 288 K. Simulated as indicated in the figure.

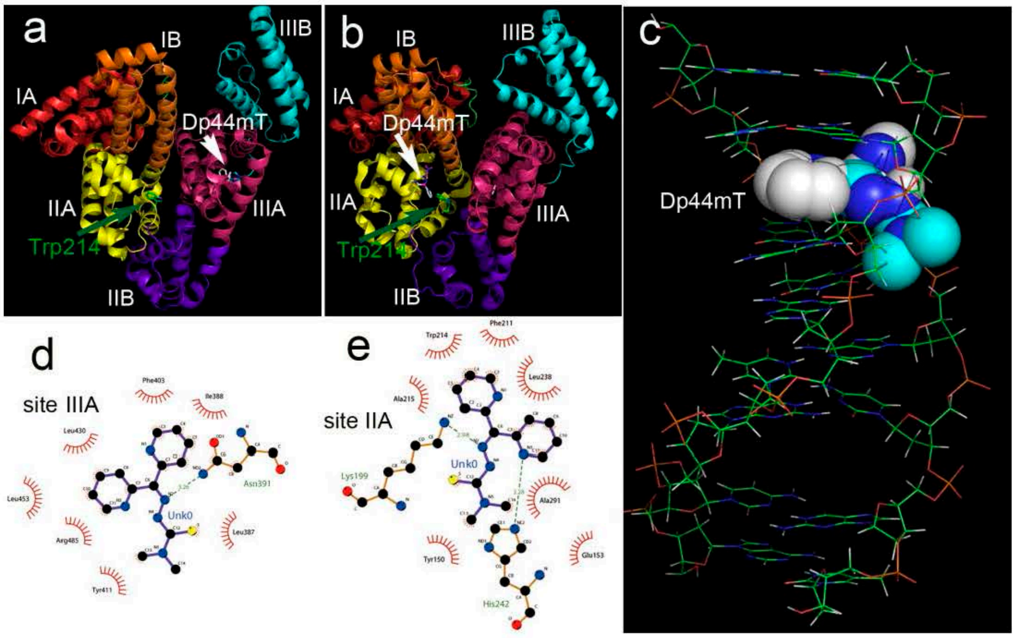

Figure S3. The interaction of Dp44mT with HAS and DNA: (**a**) Dp44mT was located in the site IIIA; (**b**) Dp44mT was located in the site IIA site; (**c**) interaction of Dp44mT with DNA; (**d**) interaction of Dp44mT with environment residues (in the figure Unk0 = Dp44mT). Dark green dash lines (**d**,**e**) indicated hydrogen bonds. The eye-like curved dark red lines represented the hydrophobic residues in contact.
